# Dietary Lithium, Silicon, and Boron: An Updated Critical Review of Their Roles in Metabolic Regulation, Neurobiology, Bone Health, and the Gut Microbiome

**DOI:** 10.3390/nu18030386

**Published:** 2026-01-24

**Authors:** Eleni Melenikioti, Eleni Pavlidou, Antonios Dakanalis, Constantinos Giaginis, Sousana K. Papadopoulou

**Affiliations:** 1 Department of Nutritional Sciences and Dietetics, School of Health Sciences, International Hellenic University, 57400 Thessaloniki, Greece; 2 Private Nutrition and Dietetics Center, 66100 Drama, Greece; 3 School of Medicine and Surgery, University of Milano-Bicocca, 20900 Monza, Italy; 4 Fondazione IRCCS San Gerardo dei Tintori, 20900 Monza, Italy; 5 Department of Food Science and Nutrition, School of Environment, University of Aegean, 81400 Myrina, Greece

**Keywords:** lithium, silicon, boron, trace elements, metabolism, oxidative stress, microbiome, bone health, neurobiology

## Abstract

Background/Objectives: Lithium (Li), silicon (Si), and boron (B) are proposed nutritional trace elements with potential roles in metabolic, neurobiological, endocrine, inflammatory, and bone-related processes. This review provides a critical synthesis of data on Li–Si–B, emphasizing (i) physiological and mechanistic pathways, (ii) human clinical relevance, (iii) shared biological domains, and (iv) safety considerations. Methods: A narrative review was conducted across PubMed, Scopus, and Web of Science from inception to January 2025. Predefined search strings targeted dietary, environmental, and supplemental exposures of lithium, silicon, or boron in relation to metabolism, endocrine function, neurobiology, inflammation, bone health, and the gut microbiome. Inclusion criteria required peer-reviewed studies in English. Data extraction followed a structured template, and evidence was stratified into human, animal, cellular, and ecological tiers. Methodological limitations were critically appraised. Results: Li, Si, and B influence overlapping molecular pathways including oxidative stress modulation, mitochondrial stability, inflammatory signaling, endocrine regulation, and epithelial/gut barrier function. Human evidence remains limited: Li is supported primarily by small trials; Si by bone-related observational studies and biomarker-oriented interventions; and B by metabolic, inflammatory, and cognitive studies of modest sample size. Convergence across elements appears in redox control, barrier function, and neuroimmune interactions, but mechanistic synergism remains hypothetical. Conclusions: Although Li–Si–B display compelling mechanistic potential, current human data are insufficient to justify dietary recommendations or supplementation. Considerable research gaps—including exposure assessment, dose–response characterization, toxicity thresholds, and controlled human trials—must be addressed before translation into public health policy.

## 1. Introduction

Trace elements influence diverse metabolic, neurological, and immunological processes. Among these, lithium (Li), silicon (Si), and boron (B) have drawn increasing interest as recent studies indicate that physiologically attainable intakes may modulate mitochondrial activity, oxidative and inflammatory pathways, extracellular matrix and collagen turnover, endocrine signaling, and gut barrier function [1,2,3,4]. Although not currently classified as essential nutrients, multiple mechanistic findings suggest that small exposures can produce measurable biological effects.

Research on Li, Si, and B has progressed largely in parallel, resulting in uneven methodological approaches and fragmented evidence. Lithium has been studied primarily in pharmacological contexts, yet ecological and translational data point to neuroprotective and metabolic effects at dietary exposure levels [5,6]. Silicon, traditionally associated with bone formation and extracellular matrix structure [7], is now understood to be highly bioavailable in the form of orthosilicic acid [8] and involved in vascular, epithelial, and metabolic regulation [9]. Boron has gained renewed attention due to its roles in mineral metabolism, Vitamin D activation, steroid hormone production [10], inflammatory cytokine modulation [4], and aspects of cognitive performance [11].

Substantial limitations, however, restrict clinical interpretation. Human data are scarce for all three elements, and Li research remains dominated by ecological designs that limit causal inference and provide insufficient control for confounding [6]. Exposure assessment is a major barrier, particularly for Si, whose concentrations in food and water vary widely and lack standardized quantification, complicating dose–response evaluation. Boron studies commonly employ heterogeneous protocols and small sample sizes, reducing statistical robustness and comparability across trials. These issues impede the establishment of toxicity thresholds [12,13,14], safe upper intake levels, and population-level exposure estimates needed for public health assessment.

Another uncertainty concerns whether Li, Si, and B influence overlapping regulatory domains. Under-investigation findings indicate potential convergence in oxidative stress modulation, inflammatory signaling, neuroendocrine pathways, and gut barrier function, but current data do not support a unified or synergistic physiological classification. This possibility remains exploratory and requires targeted investigation [15,16].

**Figure 1:**
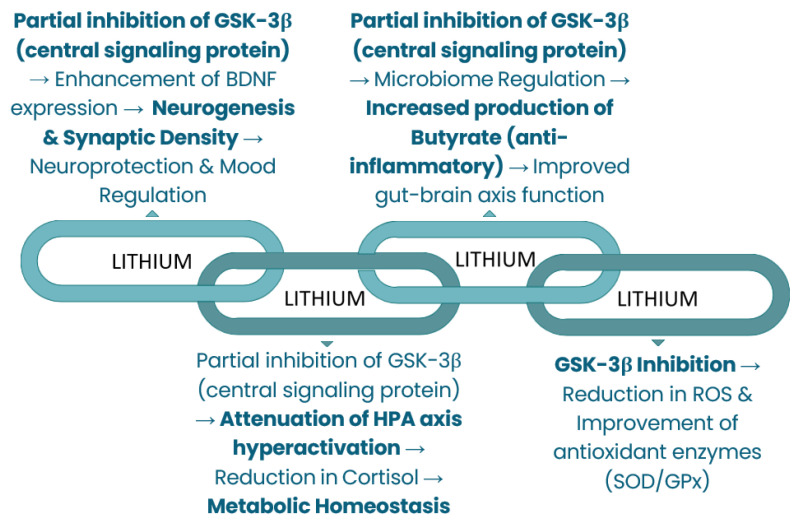
Metabolic regulations of lithium.

**Figure 2:**
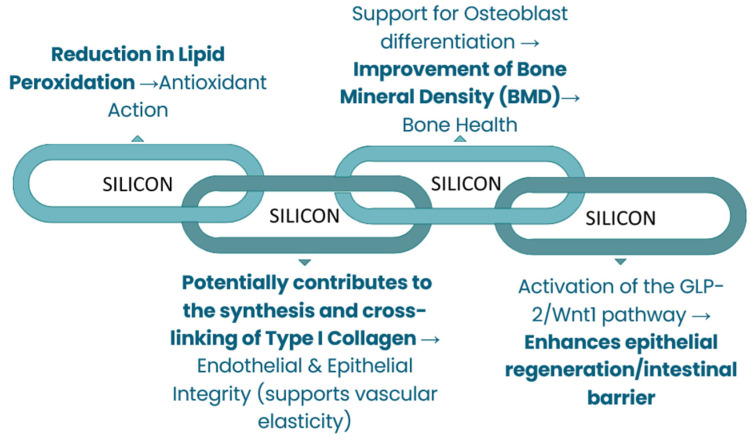
Metabolic regulations of silicon.

**Figure 3:**
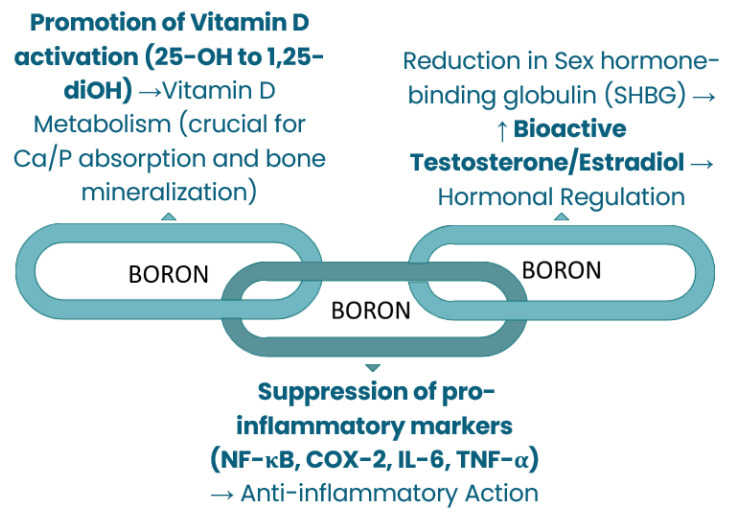
Metabolic regulations of boron.

Therefore, this review aims to firstly evaluate physiological functions of dietary Li, Si, and B (available in Figure 1, Figure 2 and Figure 3) and synthesize mechanistic and translational evidence. Simultaneously, the safety considerations provide the necessary public health context by defining natural intake variability and toxicity boundaries, while the final aim of identifying critical research gaps furnishes an explicit roadmap for future controlled human trials and targeted investigations required for eventual, responsible translation into evidence-based public health policy.

## 2. Methods

### 2.1. Data Collection

A structured search was conducted across PubMed, Scopus, and Web of Science from database inception to January 2025. In addition to this, a supplementary search was conducted in Google Scholar for citation chaining, which served to identify key articles referenced within the primary findings.

The specific keywords used in all databases were “lithium”, “trace lithium”, “dietary lithium”, “silicon”, “orthosilicic acid”, “boron”, “metabolism”, “oxidative stress”, “microbiome”, “bone”, “inflammation”, and “endocrine”. All searches were restricted to English-language studies and were further limited to publications possessing a DOI (Digital Object Identifier).

The inclusion criteria for comprehensive synthesis mandated the selection of peer-reviewed studies possessing a valid Digital Object Identifier (DOI) to ensure quality control, methodological rigor, and verifiability of the primary literature. This evidence base was exclusively drawn from human, animal, or cellular studies that specifically examined Li, Si, or B, establishing a crucial translational tiered approach. Furthermore, the selection was strictly limited to studies reporting outcomes relevant to core physiological domains, including metabolism, bone health, neurobiology, inflammation, or the gut microbiota, thereby defining the precise scope of the element’s potential systemic effects. Crucially, while existing review articles were permitted for contextual framing, they were not designated as primary evidence to maintain the integrity of the critical synthesis and ensure conclusions were based solely on original research data.

The exclusion criteria were strategically applied to ensure the synthesized evidence maintained both methodological rigor and nutritional relevance. Specifically, papers were rejected if they lacked a valid DOI or extractable data/exposure information, thereby guaranteeing the verifiability and quantitative utility of the included studies. Critically, to maintain focus on the trace element’s dietary role, the review excluded pharmacological Li trials—where doses exceed nutritional relevance—along with studies utilizing industrial silica, non-nutritional B compounds, or undefined proprietary water compositions. This comprehensive screening process ensured the final evidence synthesis was strictly limited to original, traceable research pertaining to the physiological effects of dietary Li, Si, and B [17].

### 2.2. Study Selection

Two authors independently screened all titles and abstracts for eligibility based on predefined criteria. Any discrepancies were discussed and resolved by consensus. A third author subsequently checked all articles included after full-text review to ensure consistency in study selection. 

Full texts were reviewed for extraction of the following:

- Study design;
- Sample size;
- Dose/exposure characteristics;
- Outcomes;
- Mechanistic pathways;
- Methodological limitations.

### 2.3. Article Screening Process

The search identified 2146 records. After deduplication, 1782 remained. 

Following title/abstract screening, 370 remained. Then 176 articles were eligible for full text review. Finally, 79 articles were included in this review, after careful reading.

## 3. Results

### 3.1. Dietary Exposure of the Elements and Global Intake Variability

Exposure to Li, Si, and B varies substantially across regions due to differences in soil mineral composition, agricultural practices, water mineral content (particularly distinguishing between bottled and tap sources), and inherent dietary patterns (such as reliance on grains for Si or fruits/vegetables for B). This heterogeneity across geographical regions severely complicates the process of achieving accurate, standardized population-level exposure quantification, thereby undermining reliable establishment of uniform dietary recommendations or the definition of universal safe upper intake limits for these elements [15,18,19,20,21,22]. Table 1 summarizes current exposure ranges from available datasets.

### 3.2. Lithium: Physiology, Mechanisms, and Safety

Lithium is naturally present in groundwater, plant foods, and mineral waters. Although best known for pharmacological applications, dietary lithium appears to influence mitochondrial stability, oxidative stress, neuroplasticity, and metabolic signaling at microdose exposures [24]. Evidence from ecological studies links higher water-lithium levels with lower suicide rates and improved mood stability [6], though methodological limitations restrict causal inference [18]. Beyond these associations, preclinical and population-level findings suggest that lithium may function as a broader metabolic modulator, subtly affecting energy homeostasis, endocrine regulation, and inflammatory balance even at low environmental exposures. Its pleiotropic actions appear to reflect a convergence of mitochondrial, neurotrophic, and immunometabolic pathways, making lithium a unique trace element whose biological relevance may extend further than previously assumed. Furthermore, interest in microdose lithium has grown due to growing nutritional, psychiatric, and gerontological hypotheses proposing that long-term low-level exposure may help maintain cellular resilience, though high-quality evidence is still limited. This mechanistic complexity underscores the importance of reviewing lithium physiology comprehensively to clarify where current findings are robust and where more rigorous human studies are required.

#### 3.2.1. Absorption, Distribution, and Excretion of Lithium

Dietary lithium is rapidly absorbed in the small intestine and exhibits near-complete bioavailability [25]. Distribution is similar to sodium, with accumulation in bone and soft tissues [26]. Excretion occurs primarily via the kidneys, making lithium homeostasis sensitive to hydration status and renal function [25,27].

#### 3.2.2. Mechanistic Pathways of Lithium

##### Mitochondrial and Redox Regulation

Low-dose lithium partially inhibits Glycogen synthase kinase-3 beta (GSK-3β) [28], stabilizing mitochondrial membrane potential, reducing ROS formation, and improving cellular antioxidant enzyme expression of Superoxide dismutase (SOD) and Glutathione peroxidase (GPx) [5]. These effects occur at concentrations far below pharmacological levels and contribute to improved neuronal energy metabolism, synaptic resilience, and metabolic homeostasis [29].

##### Neuroplasticity and Neuroprotection

Experimental studies consistently demonstrate that trace levels of Li enhance neuroplasticity and provide significant neuroprotection through several interconnected molecular pathways. Lithium acts as a potent inhibitor of (GSK-3β), a central regulatory enzyme, and this inhibition leads directly to the enhanced expression of Brain-Derived Neurotrophic Factor (BDNF) and subsequent promotion of hippocampal neurogenesis and synaptic density. Furthermore, these actions, alongside the modulation of CREB signaling and the stimulation of autophagy (a key cellular clearance process), confer resistance to stress-induced neuronal atrophy and reduce the hyperphosphorylation of the Tau protein, mechanisms which collectively account for the improved memory performance and reduced neuroinflammation observed in rodent studies utilizing drinking water-based lithium exposures [24,30,31].

##### Endocrine and Stress Physiology

The modulatory effects of trace Li on endocrine and stress physiology are significant, primarily manifesting as an attenuation of HPA-axis hyperactivation. Mechanistically, lithium achieves this homeostatic balance by reducing cortisol responses and normalizing glucocorticoid receptor (GR) signaling, thereby restoring the crucial negative feedback loop necessary for terminating the stress response [32,33]. Furthermore, it exerts a wider regulatory influence by modulating sympathetic–adrenal outputs, complementing its HPA-axis effects. These observed actions—which functionally enhance the body’s resilience and dampen systemic stress reactivity—provide a plausible mechanistic underpinning for the ecological associations noted between higher natural lithium levels and the observation of improved mood stability and reduced psychiatric pathology [34,35].

##### Immunological and Anti-Inflammatory Effects

Low-dose lithium reduces IL-6, IL-1β, and TNF-α through partial GSK-3β inhibition and NF-κB downregulation. It also shifts T-cell balance toward regulatory phenotypes (↑Treg, ↓Th17). This immune modulation supports both gut and systemic inflammatory homeostasis [36,37,38].

##### Microbiome Interactions

Preclinical data suggest that trace lithium modulates Microbiome Interactions by favorably altering gut microbiota composition [39], specifically by increasing butyrate-producing species and concurrently reducing pro-inflammatory taxa [40]. This compositional shift facilitates critical functional improvements, including enhanced short-chain fatty acid (SCFA) production, subsequent reinforcement of gut barrier integrity (reducing systemic exposure to inflammatory agents), and optimization of vagal signaling along the gut–brain axis. These collective effects contribute to a beneficial decrease in the systemic inflammatory tone [41]; however, while promising, the evidence establishing this link remains predominantly preclinical, necessitating validation through controlled human trials to confirm clinical translational relevance [42].

#### 3.2.3. Safety and Toxicity of Lithium

Given that lithium possesses a narrow therapeutic index at pharmacological doses (300–1200 mg/day), meaning toxicity is a significant risk at levels not far above therapeutic needs, the typical dietary exposure is fundamentally different, ranging at vastly lower levels, usually between 650 and 3100 μg/day [43]. Consequently, dietary lithium intake from water and food is generally considered safe, and official bodies like the WHO and EFSA have not established an Upper Limit (UL) for this type of exposure. Nevertheless, caution is warranted for specific groups, including individuals with impaired renal function, those who are dehydrated, and those taking certain medications like diuretics or ACE inhibitors, as they face a theoretical or heightened risk of accumulation. Ultimately, while dietary intake is safe, the supplementation of lithium cannot be recommended due to the insufficient human dose–response data and the unknown long-term safety thresholds associated with this element [23].

### 3.3. Silicon: Physiology, Mechanisms, and Safety

Silicon is the second most abundant element in the Earth’s crust and is widely distributed in plant-based foods and natural waters. In human nutrition, silicon is primarily encountered as orthosilicic acid (OSA), the soluble, monomeric, and biologically relevant form. Due to its structural role in connective tissues and its regulatory functions in several metabolic pathways, silicon is increasingly recognized as an important dietary component influencing skeletal integrity, vascular function, and gut health [44,45]. The following subsections provide an expanded overview of its absorption, physiological mechanisms, and safety considerations.

#### 3.3.1. Absorption, Distribution, and Excretion of Silicon

The pharmacokinetics of silicon demonstrate that orthosilicic acid (OSA), its nutritionally relevant form, exhibits high bioavailability at approximately 40–50%, meaning a significant portion is readily absorbed into the bloodstream [19]. This efficient absorption makes sources like mineral waters, beer, whole grains, and root vegetables particularly effective dietary contributors of bioavailable silicon [8,45]. Conversely, the text emphasizes that other, less soluble forms of the element, such as polymeric or particulate silicates, have far lower absorption rates, confirming that the chemical structure is the primary determinant of silicon’s nutritional value and subsequent biological activity within the body [2,19,46,47,48].

#### 3.3.2. Mechanistic Pathways of Silicon

##### Collagen Synthesis and Extracellular Matrix Integrity

Silicon OSA plays a critical structural and functional role within connective tissues, primarily through its involvement in Collagen Type I synthesis, cross-linking, and overall stabilization of the extracellular matrix (ECM) proteins [19,46]. A growing body of evidence indicates that bioavailable silicon supports the early stages of collagen formation by facilitating the initiation of fibril assembly and influencing enzymes involved in matrix maturation. Through these actions, silicon contributes to improved organization, density, and mechanical strength of the organic bone matrix [49].

Beyond its skeletal role, silicon’s effects on collagen synthesis extend to multiple connective tissue systems. Enhanced collagen fiber quality and cross-linking directly translate to greater resilience of tendons, ligaments, and cartilage, supporting tissue elasticity and reducing susceptibility to microdamage. In dermal tissues, adequate OSA availability is associated with improved skin firmness and structural integrity due to strengthened ECM architecture.

These ECM-related effects are also relevant for cardiovascular physiology. OSA contributes to the maintenance of vascular elasticity by supporting healthy collagen and elastin networks within arterial walls—an essential feature for buffering pulsatile pressure and maintaining stable blood flow. Reduced silicon availability has been hypothesized to accelerate vascular stiffening, whereas adequate dietary intake may help preserve arterial compliance and support long-term cardiovascular function [46].

##### Skeletal Effects and Bone Mineralization

Silicon, particularly as orthosilicic acid (OSA), is essential for bone formation and mineralization. It promotes osteoblast differentiation, supporting the maturation of precursor cells into bone-forming cells and enhancing bone matrix synthesis. Adequate OSA intake correlates with increased bone collagen content, improved trabecular architecture, and higher bone mineral density (BMD) through the structural integration of calcium, magnesium, and boron, ultimately reinforcing mechanical strength and resilience against fracture [45,48,50,51,52,53,54].

##### Vascular Structure and Metabolic Regulation

Silicon OSA contributes to vascular health primarily by maintaining the structural integrity of arterial walls. It supports the collagen and elastin networks critical for vascular elasticity, which in turn influences blood pressure regulation and cardiovascular function [49,51]. Preclinical studies indicate that OSA reduces oxidative stress and lipid peroxidation in vascular tissues, mitigating damage associated with aging and metabolic disease [3,9,20,55]. Limited human evidence suggests that silicon-rich water may also modestly improve glucose metabolism, indicating a potential role in metabolic regulation that warrants further investigation [56].

##### Gut Barrier Integrity and Epithelial Regeneration

Beyond skeletal and vascular effects, silicon OSA modulates gut epithelial health. Consumption of silicon-rich water has been shown to activate the GLP-2/Wnt1 signaling pathway, enhancing intestinal repair and epithelial growth [56,57]. This mechanism increases villus height, improves nutrient absorption, and strengthens tight-junction protein expression, collectively reinforcing gut barrier function. These effects support resilience against inflammation and early-life stress, linking OSA to gut immunity and broader metabolic homeostasis [21,58,59].

##### Safety and Toxicity of Silicon

Silicon derived from food and water, primarily in the soluble, bioavailable form of OSA, is widely regarded as safe for human consumption with no evidence of toxicity at nutritional concentrations. Crucially, it is imperative to distinguish this naturally occurring dietary OSA from industrial crystalline silica, which is a particulate respiratory toxin associated with occupational hazards like silicosis and bears no relationship to human nutrition [56]. Regulatory bodies such as the European Food Safety Authority (EFSA) and the WHO have not established an official Upper Limit (UL) for dietary OSA, largely because the excess soluble OSA is rapidly and efficiently excreted by the kidneys. Furthermore, human clinical trials demonstrate that consuming OSA through supplements (not supplemental nano-silica or formulated silicon products; those products require further investigation) at doses up to 50 mg/day—which is comparable to or slightly above the median estimated dietary intake of 20–60 mg/day—shows no adverse effects, firmly supporting its safety profile [8,12,13,60].

### 3.4. Boron: Physiology, Mechanisms, and Safety

Boron is a naturally occurring trace element distributed widely in the environment and throughout the human diet, with its richest sources found in plant-based foods such as fruits, nuts, leafy vegetables, and legumes. Drinking water also contributes meaningfully to daily intake, although concentrations vary geographically based on soil composition and groundwater mineral content [61]. Although historically overlooked in human nutrition, an expanding body of biochemical and clinical research demonstrates that boron exerts a range of regulatory functions affecting bone metabolism [22,62], inflammatory signaling [63], endocrine activity, and neurocognitive performance. These findings have positioned boron as a biologically relevant micronutrient contributing to metabolic resilience and overall health [64,65].

#### 3.4.1. Absorption, Distribution, and Bioavailability

Boron exhibits a highly efficient absorption profile in the human body, with bioavailability typically exceeding 85%, where it is primarily absorbed across the gastrointestinal tract as the un-ionized molecule, boric acid (H3BO3) [22,62]. Once absorbed, boron is rapidly distributed throughout the body’s water and is maintained in a steady-state equilibrium; however, excess intake is quickly managed due to its short half-life, being eliminated efficiently and almost completely via renal excretion as boric acid [2]. Consequently, the actual daily intake of boron varies widely, typically ranging from 0.5 to 3 mg/day, a range that is strongly dependent upon an individual’s consumption of boron-rich foods, particularly fruits and vegetables, highlighting the direct link between dietary choices and the concentration of this trace element in the body [64,66,67,68].

#### 3.4.2. Mechanistic Pathways of Boron

##### Vitamin D and Mineral Metabolism

Boron is a key metabolic modulator that significantly influences the bioavailability and functional efficacy of other essential micronutrients, particularly those governing bone health. This trace element enhances the activation of Vitamin D by promoting the conversion of 25-hydroxyvitamin D to its most active hormonal form, 1,25-dihydroxyvitamin D, thereby amplifying its crucial role in calcium homeostasis [2,69]. Furthermore, boron exhibits a clear regulatory effect on mineral retention, specifically increasing magnesium retention within the body and reducing its urinary excretion, which is vital as magnesium is a necessary cofactor for hundreds of enzymatic reactions, including those related to energy metabolism [65,69]. This retention mechanism, coupled with the enhanced efficacy of activated Vitamin D, leads to improved calcium utilization, ensuring that calcium is effectively incorporated into the bone matrix, with all these effects collectively supporting robust bone health and overall metabolic flexibility [4,62,69].

##### Inflammatory and Oxidative Modulation

Boron acts as an essential modulator of inflammatory and oxidative stress pathways by directly influencing key signaling molecules within the body. Specifically, boron has been shown to downregulate central inflammatory mediators, including NF-κB (Nuclear Factor kappa B), which controls the transcription of pro-inflammatory genes, as well as the expression of COX-2 (Cyclooxygenase-2), a critical enzyme in prostaglandin production [4]. This inhibitory action extends to the reduction in circulating pro-inflammatory cytokines such as Interleukin-6 (IL-6) and Tumor Necrosis Factor-alpha (TNF-α), effectively dampening the overall inflammatory tone [63,66]. In addition, boric acid inhibits adipogenesis by downregulating adipogenesis-related genes and proteins, including CCAAT-enhancer-binding protein α and peroxisome proliferator-activated receptor γ, through modulation of key growth factors and the β-catenin, AKT, and extracellular signal-regulated kinase (ERK) signaling pathways, while suppressing mitotic clonal expansion without inducing apoptosis, thereby further supporting its role in metabolic regulation [70]. Conversely, numerous studies in both animal models and humans demonstrate that boron deficiency leads to an increased susceptibility to inflammation and elevated markers of oxidative stress, underscoring boron’s necessity in maintaining cellular redox homeostasis and promoting overall health and metabolic resilience [62,63,66,68,71].

##### Hormonal Regulation

Boron significantly influences steroid hormone metabolism, functioning as a key regulator that affects the bioavailability of both male and female sex hormones. This trace element has been shown in human studies to increase the levels of bioactive testosterone and estradiol, essential hormones for muscle function and bone density, respectively, by effectively reducing the concentration of Sex Hormone-Binding Globulin (SHBG); this frees up bound hormones, making them biologically active and readily available to target tissues [10,71]. Furthermore, boron exhibits a modulatory effect on cortisol dynamics, potentially aiding in the body’s stress response and reducing the catabolic effects associated with chronic stress, which ultimately has profound implications for optimizing metabolism, supporting muscle function, and enhancing overall cognitive performance [2,4]. Evidence also suggests that boron exposure is associated with alterations in thyroid hormone levels, thyroid volume, and structure—possibly due to boron accumulation in the thyroid and potential competition with iodine—although further clinical and experimental studies are needed to clarify its role in hypothyroidism and goiter, particularly in regions with high environmental boron levels [72].

##### Neurological and Cognitive Effects

Boron has been proposed to influence neurological function, but the available evidence is derived primarily from older, small-scale human trials [11,73,74,75]. These studies report that low boron intake is associated with alterations in electrophysiological brain activity—such as shifts in EEG patterns—and modest decrements in attention, short-term memory, and psychomotor performance [75]. Participants consuming low boron diets often show reduced accuracy and slower response times on tasks requiring immediate memory retrieval and fine motor coordination. However, these findings should be interpreted cautiously, as the studies relied on dated EEG techniques, limited sample sizes, and insufficient control for micronutrient cofactors that also affect cognitive function. Although some supplementation trials have suggested improvements in cognitive and sensorimotor performance with optimized boron intake, the absence of rigorous replication using contemporary methods limits confidence in the magnitude and generalizability of these effects [4,11,65,76]. Furthermore, B influences central nervous system function by modulating blood–brain barrier permeability and promoting boron accumulation in the brain [77,78] and potentially B has been identified as facilitating the release of the TDP43 protein, which is associated with DNA methylation. This could contribute to the advancement of cell preservation and repair strategies in the context of neuroprotection [78].

#### 3.4.3. Safety, Toxicity, and Regulatory Considerations

While boron intake from typical dietary sources (estimated at 1–3 mg/day) and water is considered safe and far below toxic thresholds, regulatory bodies have established specific Upper Limits (ULs) due to evidence of toxicity at higher concentrations. The European Food Safety Authority (EFSA) sets the UL for adults at 10 mg/day, while the World Health Organization (WHO) has provided guidance suggesting a tolerable upper intake in the range of 10–20 mg/day [13,60]. Acute, excessive boron intake, typically exceeding 20 mg/day over extended periods, may lead to clinical manifestations such as mild gastrointestinal distress and dermatitis [61,64]. More importantly, animal models have demonstrated reproductive toxicity at extremely high exposure levels, leading experts to advise caution; thus, despite its beneficial physiological roles, routine boron supplementation is generally not recommended due to limited long-term human safety evidence near the established ULs [13,22,60,62].

### 3.5. Integrative Mechanistic Model: Converging Pathways of Lithium, Silicon, and Boron

Although lithium, silicon, and boron have historically been considered unrelated nutritionally, emerging evidence reveals functional convergence across multiple biological systems. These overlaps are not indicative of proven synergy, but they suggest a hypothesis-generating model in which Li–Si–B jointly influence metabolic, neuroimmune, skeletal, and gastrointestinal functions [15,16].

#### 3.5.1. Oxidative and Inflammatory Crosstalk

The three trace elements—Li, Si, B—demonstrate a powerful functional convergence in reducing systemic redox burden and inflammatory signaling, although each operates through a distinct, yet complementary, molecular pathway. Lithium initiates its anti-inflammatory and antioxidant effect by inhibiting Glycogen Synthase Kinase-3 beta (GSK-3β), a central enzyme whose suppression reduces the production of Reactive Oxygen Species (ROS) and enhances mitochondrial stability [5]. Concurrently, silicon acts at the level of cellular defense and damage control, evidenced by its role in decreasing lipid peroxidation and upregulating intrinsic antioxidant defenses, such as glutathione [3,9]. Finally, boron targets the initiation of the inflammatory cascade, significantly downregulating the activation of the master transcription factor NF-κB and consequently reducing the expression of critical pro-inflammatory cytokines like IL-6 and TNF-α [63,66]. Because all three elements converge on inflammation-related pathways, combined exposure may theoretically reduce systemic redox burden more effectively than individual elements. However, no studies have directly tested Li–Si–B co-exposure, and any synergistic interpretation remains speculative.

#### 3.5.2. Endocrine and Neuroendocrine Interactions

The synergistic effects of Li, B, and Si create a converging influence on endocrine and neuroendocrine stability, collectively working to optimize metabolic homeostasis and reduce stress load. Specifically, lithium is crucial for modulating the Hypothalamic–Pituitary–Adrenal (HPA) axis activity, a primary stress pathway, effectively mitigating stress-induced cortisol elevations and promoting mental resilience [5]. Complementarily, boron acts primarily on steroidogenesis and vitamin metabolism, influencing sex hormones like testosterone and estradiol, enhancing the activation of Vitamin D, and supporting magnesium homeostasis [10,65,69]. Finally, silicon supports essential mineral–hormone interactions—for instance, in regulating the effects of parathyroid hormone (PTH) and Vitamin D on bone and collagen synthesis—thereby ensuring the integrity of the structural systems that hormones regulate [15,16,69]. The aggregated effects of Li, Si, and B may theoretically contribute to broader metabolic or neuroendocrine stability; however, direct synergistic or interactive effects have not been demonstrated, and proposed integration of these mechanisms remains conceptual.

#### 3.5.3. Microbiome-Driven Mechanisms

Li, Si, and B converge to reinforce gut health by leveraging distinct mechanisms that enhance barrier integrity and modulate the gut microbiome, collectively lowering systemic inflammation. Li exerts its influence by restructuring the microbial composition, promoting the growth of beneficial, butyrate-producing bacteria while simultaneously modulating pro-inflammatory microbial taxa [5]. Si, acting on the host side, directly enhances epithelial regeneration and repair mechanisms by activating the GLP-2/Wnt pathways, thereby physically strengthening the intestinal lining [52]. Meanwhile, B plays a role in stabilizing beneficial microbial communities and may influence quorum-sensing processes, which bacteria use for group coordination and virulence [4,15,59,62,74]. The complementary actions of the three elements may collectively promote short-chain fatty acid production, limit microbial translocation, and lower systemic low-grade inflammation. These potential interactions, however, are inferred rather than empirically validated, and require dedicated experimental evaluation.

#### 3.5.4. Bone–Gut–Brain Axis

The proposed functional convergence of Li, Si, and B establishes a novel hypothesis concerning the optimization of the complex Bone–Gut–Brain axis, where the distinct actions of each element intersect at critical regulatory points. Si and B exert direct influence over bone metabolism and matrix integrity, with silicon enhancing collagen synthesis and boron regulating Vitamin D and mineral homeostasis; simultaneously, both elements modulate inflammatory tone by suppressing key cytokines, a shared communication pathway across the axis [4,45]. Conversely, Li primarily targets the central nervous system, enhancing neuroplasticity and regulating microbiome composition, which directly impacts gut-derived neuroendocrine signals [5]. The combined, systemic impact of these elements—spanning enhanced nutrient absorption (via Si and B), strengthened endocrine regulation (via Li and B), and a reduction in inflammatory tone (via all three)—suggests that adequate Li–Si–B exposure may contribute to optimized Bone–Gut–Brain communication, though causal synergy remains unproven and requires further dedicated investigation [15,16,59,74,75].

## 4. Evidence Strength, Research Gaps, and Critical Appraisal

A central objective of this review is to provide a transparent hierarchy of evidence. The following subsections evaluate the robustness, limitations, and methodological gaps across Li, Si, and B (Table 2).

For Li, the strongest evidence lies in ecological studies correlating water concentration with suicide rates, though the critical caveat remains that such designs cannot infer causality, given the high sensitivity to socioeconomic and geographic confounding [5].

Si shows its most robust findings in observational data on bone health [45,50,51,52,54], with emerging evidence on gut barrier function [59], yet research is hampered by inconsistent dietary intake estimation due to high variability in food content [8,19]. The safety of silicon compounds has been addressed by regulatory bodies [12].

B is best supported by controlled trials demonstrating effects on inflammation [63,66] and mineral metabolism [68,71], but suffers from small sample sizes and heterogeneous endpoints in human studies, including those on brain function [77,78]. Shared methodological weaknesses across all three include high ecological confounding, poor dose–response characterization within nutritional ranges [22], largely preclinical microbiome data, and inadequate safety reporting [13,19], making several nutritional thresholds for benefit/toxicity currently undefined.

Consequently, research priorities must focus on generating standardized global exposure databases, conducting large-scale dose–response human trials, establishing clarified ULs and toxicity thresholds, and performing multi-omics microbiome integrations to fully validate their mechanistic interactions and clinical relevance [15,16].

## 5. Public Health Implications

The population-level impacts of Li, Si, and B are profoundly influenced by significant geographic variability in environmental exposure, demanding careful consideration for public health planning. The intake of these trace elements is highly diverse: Li concentrations can vary by over 100-fold between groundwater sources, mirroring local geological conditions. Similarly, Si exposure depends heavily on the consumption of specific mineral waters and cereal intake, while B levels are intrinsically linked to soil composition and the regional consumption patterns of fruits and vegetables [4,70,74,76].

### 5.1. Potential Health-Relevant Variability

This exposure heterogeneity is associated with observable health trends [5]. Specifically, regions characterized by high natural Si intake often exhibit a lower prevalence of bone fractures, supporting its role in skeletal health. Analogously, areas reporting higher natural Li in their water sources show an associative link with lower community suicide rates; however, it is critical to emphasize that this is an ecological association and does not imply causality. Furthermore, populations with adequate B exposure generally display improved inflammatory and cognitive profiles due to its known regulatory roles [11].

### 5.2. No General Recommendation for Supplementation

Despite the compelling mechanistic data and the suggestive ecological findings, the current body of evidence does not support routine supplementation of Li, Si, or B for the general population. Health agencies have not established Required Intakes (RIs) for any of these elements, and significant safety uncertainties remain, especially concerning the long-term effects of chronic, high-dose intake [12]. Nevertheless, certain population subgroups are potentially more affected by marginal deficiencies, including the elderly with low bone density (Si, B), individuals with poor dietary diversity (Si, B deficiencies), and—tentatively, based on non-causal evidence—populations residing in low-lithium water regions [45].

### 5.3. Water Policy Considerations

While current evidence does not justify modifying municipal lithium concentrations for health purposes, several additional considerations merit attention. Population-wide changes to Li exposure could introduce unintended consequences, as lithium has a relatively narrow therapeutic margin in pharmacological settings and even modest increases in baseline intake may pose risks for vulnerable groups, including individuals with renal or thyroid disorders or those using lithium-containing medications. Altering water composition may also interact with local geochemistry, treatment processes, and mineral profiles, complicating monitoring and risk assessment.

Ethical considerations are central to any population-level modification of essential resources. Adjusting trace element content in public drinking water constitutes involuntary exposure and therefore requires robust causal evidence of benefit, minimal risk, and broad social consensus—criteria not yet met for lithium.

Regulatory precedents further underscore the need for caution. Policies concerning fluoride, iodine, selenium, and arsenic demonstrate that modifications to municipal water composition are implemented only when supported by extensive toxicological characterization and long-term epidemiological data. Accordingly, systematic surveillance of trace-element concentrations, rather than alteration, represents the most appropriate public health approach at present [15,16,18,56,58,67].

The comparative and functional findings are summarized in Table 3. This synopsis highlights the convergence of the three elements across key biological domains, demonstrating that while Li, Si, and B employ distinct molecular pathways, their collective physiological output often concerns the modulation of oxidative stress, neuroendocrine balance, and structural integrity. It is essential to note, however, that the disparity in the strength of human evidence—ranging from ecological association for Li to observational and small-scale trials for Si and B—critically limits the ability to draw firm, causal conclusions regarding supplementation or dietary recommendations.

## 6. Conclusions

Trace elements exert subtle yet functionally significant influences on metabolic, neurological, and immunological physiology. Among these, lithium (Li), silicon (Si), and boron (B) have gained renewed attention as emerging evidence indicates that physiologically relevant intakes can modulate mitochondrial activity, oxidative and inflammatory pathways, collagen and extracellular matrix dynamics, endocrine regulation, and gut barrier integrity [1,2,3,4]. Although these elements are not currently classified as essential nutrients, converging mechanistic data suggest that even low-level exposures may produce measurable biological effects.

Research on Li, Si, and B has traditionally progressed within separate disciplinary domains, yielding a fragmented and methodologically heterogeneous evidence base. Lithium has been examined predominantly as a pharmacological agent, yet ecological and translational observations indicate that lower dietary exposures may also exert neuroprotective and metabolic effects [5,6]. Silicon, historically associated with skeletal development and extracellular matrix structure [7], has more recently been characterized as highly bioavailable in its orthosilicic acid form [8] and potentially relevant to vascular, epithelial, and metabolic function [9]. Boron has similarly re-emerged as a nutrient of interest due to its roles in mineral metabolism, Vitamin D activation, steroid hormone regulation [10], inflammatory cytokine modulation [4], and cognitive processes [11].

Despite this expanded interest, substantial limitations continue to constrain interpretation as essential elements. Human studies remain limited across all three elements, and Li research is still dominated by ecological designs that restrict causal inference and limit adjustment for confounding. Exposure assessment further complicates evaluation—particularly for Si—due to its variable and insufficiently standardized distribution in food and water, which precludes robust dose–response characterization. Boron investigations are also challenged by inconsistent study protocols and frequent reliance on small, underpowered intervention trials, reducing both statistical reliability and inter-study comparability. These constraints hinder the establishment of toxicity thresholds [12,13,14], safe upper intake levels, and population-level exposure estimates that are necessary for evidence-based public health recommendations.

An additional conceptual uncertainty concerns the possibility that Li, Si, and B influence overlapping mechanistic domains—such as oxidative stress, inflammation, neuroendocrine signaling, and gut barrier function. While preliminary findings suggest potential points of convergence, current evidence does not support a unified physiological classification. Any integrative interpretation should therefore be considered hypothesis-generating rather than conclusive [15,16,46,62].

Finally, this study aims to identify critical research gaps and establish priorities for future human studies, providing a roadmap for rigorous investigations to clarify whether Li, Si, and B have actionable roles in metabolic regulation, neurobiology, and public health, and to inform the development of evidence-based nutritional and policy guidance.

## Figures and Tables

**Table 1:** Estimated dietary and water-derived intakes of lithium, silicon, and boron by region [4,9,15,18,20,21,22,23].

Region	Lithium Intake (μg/day)	Silicon Intake (mg/day)	Boron Intake (mg/day)	Major Sources	Notes on Bioavailability
Northern Europe	10–150	20–50	1–3	Mineral waters, grains, vegetables	Orthosilicic acid highly bioavailable
Southern Europe	5-80	30-60	1–4	Mineral waters, wine, fruits	Boron bioavailability high from plant foods
North America	5–150	20–55	0.8–2	Drinking water, cereals	Lithium varies > 100 μg by region
Asia	10–300	15–40	1–3	Rice, vegetables	High variation due to hydrogeology
Australia/NZ	5–100	15–45	1–3	Drinking water, fruits	Limited nationwide datasets

**Table 2:** Evidence strength and key limitations for lithium, silicon, and boron.

Element	Human Evidence	Animal Evidence	In Vitro Evidence	Overall Strength	Key Limitations
Lithium (Li)	Ecological/observational: inverse association between water Li and suicide rates [5]	Strong neuroprotective and anti-inflammatory effects at physiological doses [5]	Suppression of inflammatory/apoptotic pathways; mechanistic plausibility [5]	Moderate	Human studies are ecological; causality cannot be inferred; limited intervention trials
Silicon (Si)	Observational: bone density; suggesting gut barrier evidence [45,46,48,54]	Collagen synthesis, bone matrix formation, intestinal barrier repair [45,46,54]	Enhanced collagen synthesis and epithelial repair via Wnt/GLP-2 [52]	Moderate	Dietary intake variability; limited controlled trials; translation from animal dosing uncertain
Boron (B)	Small controlled trials: inflammation and mineral metabolism; heterogeneous neurocognitive outcomes [10,11,57,59,60]	Consistent effects on bone metabolism, inflammation, steroid hormone regulation [4,45]	NF-κB inhibition, cytokine modulation, mineralization pathways [4,15,16]	Moderate	Small sample sizes; heterogeneous endpoints; limited long-term data
Shared Considerations	High ecological confounding; poorly characterized dose–response	Mostly preclinical; variable translational relevance	Limited human validation	–	Inconsistent safety reporting; undefined benefit/toxicity thresholds; largely preclinical microbiome data

**Table 3:** Functional convergence of trace elements Li, Si, and B.

Element	Primary Physiological Roles	Key Mechanisms of Action	Strength of Human Evidence and Core Findings	Citations
Lithium (Li)	Neuroprotection, Mood Regulation, Metabolic Homeostasis.	Partial inhibition of GSK-3β. Enhanced BDNF expression. Attenuation of HPA-axis hyperactivation.	Limited: Primarily ecological studies. Requires controlled human trials.	[5,6,24,32,34]
Silicon (Si)	Bone Health, Collagen Synthesis, Vascular and Epithelial Integrity.	Essential for Type I Collagen synthesis and cross-linking. Supports Osteoblast differentiation. Activation of GLP-2/Wnt1 pathway for intestinal repair.	Moderate: Strongest findings in observational studies on Bone Mineral Density (BMD). Supported by biomarker-oriented interventions.	[7,19,46,49,52]
Boron (B)	Vitamin D Metabolism, Hormonal Regulation, Anti-inflammatory Action, Neuroprotective	Promotes Vitamin D activation Reduces SHBG (Sex Hormone-Binding Globulin). Suppression of NF-kappa B and COX-2 inflammatory markers. Prevention, regulation, or treatment of neurodegeneration and of the onset and progression of Alzheimer’s disease.	Moderate: Supported by controlled trials for inflammation and hormonal metabolism. Suffers from small sample size and heterogeneous endpoints.	[2,4,10,57,59]

## Data Availability

No new data were created or analyzed in this study. Data sharing is not applicable to this article.
